# Is cancer stage data missing completely at random? A report from a large population-based cohort of non-small cell lung cancer

**DOI:** 10.3389/fonc.2023.1146053

**Published:** 2023-04-04

**Authors:** Andrew G. Robinson, Paul Nguyen, Catherine L. Goldie, Matthew Jalink, Timothy P. Hanna

**Affiliations:** ^1^ Division of Cancer Care and Epidemiology, Queen’s Cancer Research Institute, Kingston, ON, Canada; ^2^ Department of Oncology, Queen’s University, Kingston, ON, Canada; ^3^ ICES, Queen’s University, Kingston, ON, Canada; ^4^ School of Nursing, Queen’s University, Kingston, ON, Canada; ^5^ Department of Public Health Sciences, Queen’s University, Kingston, ON, Canada

**Keywords:** missing data, non-small cell lung cancer, administrative data, population-based, cancer stage

## Abstract

**Introduction:**

Population-based datasets are often used to estimate changes in utilization or outcomes of novel therapies. Inclusion or exclusion of unstaged patients may impact on interpretation of these studies.

**Methods:**

A large population-based dataset in Ontario, Canada of non-small cell lung cancer patients was examined to evaluate the characteristics and outcomes of unstaged patients compared to staged patients. Multivariable Poisson regression was used to evaluate differences in patient-level characteristics between groups. Kaplan-Meier estimates of survival and log-rank statistics were utilized.

**Results:**

In our Ontario cohort of 51,152 patients with NSCLC, 11.2% (n=5,707) were unstaged, and there was evidence that stage data was not missing completely at random. Those without assigned stage were more likely than staged patients to be older (RR [95%CI]), (70-79 vs. 20-59: 1.51 [1.38-1.66]; 80+ vs. 20-59: 2.87 [2.62-3.15]), have a higher comorbidity index (Score 1-2 vs 0: 1.19 [1.12-1.27]; 3 vs. 0: 1.49 [1.38-1.60]), and have a lower socioeconomic class (4 vs. 1 (lowest): 0.91 [0.84-0.98]; 5 vs. 1 (lowest): 0.89 [0.83-0.97]). Overall survival of unstaged patients suggested a mixture of early and advanced stage, but with a large proportion that are probably stage IV patients with more rapid death than those with reported stage IV disease.

**Conclusion:**

In this case study, evaluation of stage-specific health care utilization and outcomes for staged patients with stage IV disease at the population level may have a bias as a distinct subset of stage IV patients with rapid death are likely among those without a documented stage in administrative data.

## Introduction

Population-based data are often used to explore stage-based outcomes of large groups of patients, and to describe treatment utilization rates for these groups in routine practice (1). However, many databases may be incomplete with less than 100% capture of variables such as stage (2).

Understanding the impact of missing stage information on studies estimating health care utilization or population-based outcomes in cancer patient data sets may be useful in interpreting various methods of estimating these rates and outcomes. Some databases may be missing stage information due to uniformly incomplete data collection of staged patients, while others may be missing stage information if patients are unstaged for medical reasons such as advanced rapidly progressive disease not amenable to active treatment. The latter condition represents data missing not completely at random, where the variable distribution (in this case stage) is different. Missing data in this case may be informative (3). If the act of being staged is associated with being ‘fit’ enough to receive treatment, then studies examining associated utilization rates or outcomes limited to patients with advanced disease with stage information may produce biased estimates compared to the true population value.

Here we provide a case study exploring patient characteristics and survival of patients stratified according to the presence of stage data. Given the high incidence and mortality of lung cancer, we explored this in a population-based sample of patients with non-small cell lung cancer (NSCLC) in the Canadian province of Ontario.

## Methods

### Study Design and Population

A population-based cohort of patients from the Ontario Cancer Registry (OCR) diagnosed with NSCLC between January 1, 2007, and December 31, 2016, were included. Ontario has a single-payer universal health care system with a population of over 14 million. We included patients with only one NSCLC diagnosis, with no history of previous chemotherapy, radiation therapy or surgery treatments. Patients were required to have a minimum of 5 years of continuous health insurance coverage prior to diagnosis to provide sufficient look back for comorbidity scoring, to be 20 years of age or older, and have a place of residence in Ontario. This study was approved by the Queen’s University Health Sciences and Affiliated Teaching Hospitals Research Ethics Board.

### Data sources

ICES is an independent, non-profit research institute whose legal status under Ontario’s health information privacy law allows it to collect and analyze health care and demographic data, without consent, for health system evaluation and improvement. These datasets were linked using unique encoded identifiers and analyzed at ICES.

### Classification of independent variables

Stage was assigned on available data from Collaborative Stage in OCR and pathological/clinical stage in the Activity Level Reporting (ALR) data. This uses information derived from clinic-reported stage and manual chart review to assign stage based on the most reliable information (e.g. chart review data may be used in priority over cancer centre reported stage). Patient demographic data at the time of diagnosis were obtained from Ministry of Health administrative data. Comorbidity was assigned based on the Elixhauser comorbidity index (a validated algorithm to classify comorbidity using International Classification of Disease codes in administrative data) with a five-year lookback with Canadian Institute for Health Information Discharge Abstract Database (DAD) and Same Day Surgery (SDS) data (4). Diagnostic codes for lymphoma, metastatic cancer and solid tumours without metastasis were not included in the score. Neighbourhood income quintile was utilized as an area-level measure of socioeconomic status. Categorization of place of residence as urban, sub-urban or rural was based on the 2008 Rurality Index for Ontario (5). Chronic diseases (e.g., asthma and congestive heart failure) were identified with ICES-derived datasets based validated algorithms.

### Classification of dependent variables

Overall survival and cancer-specific survival were measured from the date of diagnosis. Follow-up data were censored at 4 years for overall survival and 2 years for cancer-specific survival. Follow-up was shorter for cancer-specific survival as cause-specific death information from Ontario’s Office of the Registrar General-Death (ORGD) is complete only up to December 31, 2018.

### Statistical analyses

Demographic and general health data were summarized by stage (including unstaged information). Multivariable Poisson regression was used to evaluate the differences in the patient-level characteristics between the unstaged and staged groups. Kaplan-Meier estimates of survival were determined according to stage. Log-rank statistics were utilized. All analyses were performed using the SAS software 9.4 (SAS Institute, Cary NC).

## Results

Of 51,152 NSCLC patients, 11.2% (5,707) were unstaged (**Table 1**). Unstaged patients were significantly more likely to be older (Relative Risk (RR) [95% Confidence Interval (CI)]: 70-79 vs. 20-59, 1.51 [1.38-1.66]; 80+ vs. 20-59, 2.87 [2.62-3.15]), reside in lower income neighbourhoods (RR [95% CI]: 4^th^ vs. 1^st^ quintile, 0.91 [0.84-0.98]; 5^th^ vs. 1^st^ quintile, 0.89 [0.83-0.97]) and rural areas (RR [95% CI]: urban vs. rural, 0.58 [0.54-0.61]; sub-urban vs. rural, 0.71 [0.66-0.77]), and have a higher comorbidity index (RR [95% CI]: 1-2 vs. 0, 1.19 [1.12-1.27]; 3+ vs. 0, 1.49 [1.38-1.60]) (**Table 2**). The occurrence of missing stage also changed over time, becoming increasingly less likely during the study period (RR year of diagnosis, per 1-year increase [95% CI]: 0.92 [0.91-0.92]). Among the unstaged group, 89.4% (5,102) died within 4 years from diagnosis. Earlier stage patients at diagnosis (stage I/II/III) comprised ~32.8% of deaths.

**Table 1:** Demographic and general health characteristics for non-small cell lung cancer (NSCLC) patients in 2007-2016.

Patient Characteristics	Best Stage Information					
	I	II	III	IV	Unstaged	Total
	N=7,959	N=3,309	N=9,967	N=24,210	N=5,707	N=51,152
Year of diagnosis						
Mean ± SD	2011.84 ± 2.91	2011.87 ± 2.71	2011.21 ± 2.91	2011.55 ± 2.75	2010.71 ± 3.29	2011.46 ± 2.89
Median (IQR)	2012 (2009-2014)	2012 (2010-2014)	2011 (2009-2014)	2012 (2009-2014)	2009 (2008-2014)	2011 (2009-2014)
Age						
Mean ± SD	70.71 ± 10.13	70.27 ± 10.07	69.71 ± 10.60	69.58 ± 11.07	75.21 ± 11.33	70.45 ± 10.94
Median (IQR)	71 (64-78)	71 (63-78)	70 (62-78)	70 (62-78)	77 (68-84)	71 (63-79)
Age (categorized)						
20-59	1,117 (14.03%)	517 (15.62%)	1,779 (17.85%)	4,673 (19.30%)	587 (10.29%)	8,673 (16.96%)
60-69	2,292 (28.80%)	977 (29.53%)	2,942 (29.52%)	7,126 (29.43%)	1,043 (18.28%)	14,380 (28.11%)
70-79	2,905 (36.50%)	1,181 (35.69%)	3,322 (33.33%)	7,474 (30.87%)	1,782 (31.22%)	16,664 (32.58%)
80+	1,645 (20.67%)	634 (19.16%)	1,924 (19.30%)	4,937 (20.39%)	2,295 (40.21%)	11,435 (22.35%)
Sex						
Female	4,345 (54.59%)	1,535 (46.39%)	4,625 (46.40%)	11,258 (46.50%)	2,781 (48.73%)	24,544 (47.98%)
Male	3,614 (45.41%)	1,774 (53.61%)	5,342 (53.60%)	12,952 (53.50%)	2,926 (51.27%)	26,608 (52.02%)
Neighbourhood income quintile						
Missing	24 (0.30%)	9 (0.27%)	31 (0.31%)	93 (0.38%)	37 (0.65%)	194 (0.38%)
1 (Lowest)	1,888 (23.72%)	841 (25.42%)	2,510 (25.18%)	5,754 (23.77%)	1,450 (25.41%)	12,443 (24.33%)
2	1,762 (22.14%)	720 (21.76%)	2,236 (22.43%)	5,411 (22.35%)	1,258 (22.04%)	11,387 (22.26%)
3	1,535 (19.29%)	662 (20.01%)	1,930 (19.36%)	4,719 (19.49%)	1,124 (19.70%)	9,970 (19.49%)
4	1,452 (18.24%)	595 (17.98%)	1,722 (17.28%)	4,430 (18.30%)	975 (17.08%)	9,174 (17.93%)
5 (Highest)	1,298 (16.31%)	482 (14.57%)	1,538 (15.43%)	3,803 (15.71%)	863 (15.12%)	7,984 (15.61%)
Urban/rural residence						
NA/Missing	82 (1.03%)	33 (1.00%)	132 (1.32%)	287 (1.19%)	121 (2.12%)	655 (1.28%)
Urban (RIO<10)	4,994 (62.75%)	1,957 (59.14%)	5,972 (59.92%)	15,584 (64.37%)	3,117 (54.62%)	31,624 (61.82%)
Sub-urban (10≤RIO<40)	2,036 (25.58%)	875 (26.44%)	2,655 (26.64%)	5,923 (24.47%)	1,506 (26.39%)	12,995 (25.40%)
Rural (40≤RIO)	847 (10.64%)	444 (13.42%)	1,208 (12.12%)	2,416 (9.98%)	963 (16.87%)	5,878 (11.49%)
Place of residence						
Erie St. Clair	412 (5.18%)	188 (5.68%)	653 (6.55%)	1,537 (6.35%)	376 (6.59%)	3,166 (6.19%)
South West	546 (6.86%)	268 (8.10%)	893 (8.96%)	1,968 (8.13%)	517 (9.06%)	4,192 (8.20%)
Waterloo Wellington	311 (3.91%)	158 (4.77%)	474 (4.76%)	1,250 (5.16%)	264 (4.63%)	2,457 (4.80%)
Hamilton Niagara Haldimand Brant	996 (12.51%)	427 (12.90%)	1,344 (13.48%)	3,331 (13.76%)	698 (12.23%)	6,796 (13.29%)
Central West	313 (3.93%)	114 (3.45%)	356 (3.57%)	959 (3.96%)	208 (3.64%)	1,950 (3.81%)
Mississauga Halton	466 (5.86%)	183 (5.53%)	525 (5.27%)	1,406 (5.81%)	327 (5.73%)	2,907 (5.68%)
Toronto Central	626 (7.87%)	235 (7.10%)	605 (6.07%)	1,788 (7.39%)	333 (5.83%)	3,587 (7.01%)
Central	785 (9.86%)	242 (7.31%)	778 (7.81%)	2,336 (9.65%)	433 (7.59%)	4,574 (8.94%)
Central East	983 (12.35%)	394 (11.91%)	1,171 (11.75%)	2,936 (12.13%)	765 (13.40%)	6,249 (12.22%)
South East	459 (5.77%)	177 (5.35%)	599 (6.01%)	1,418 (5.86%)	322 (5.64%)	2,975 (5.82%)
Champlain	984 (12.36%)	388 (11.73%)	1,170 (11.74%)	2,151 (8.88%)	481 (8.43%)	5,174 (10.11%)
North Simcoe Muskoka	348 (4.37%)	171 (5.17%)	425 (4.26%)	1,025 (4.23%)	309 (5.41%)	2,278 (4.45%)
North East	541 (6.80%)	271 (8.19%)	766 (7.69%)	1,563 (6.46%)	458 (8.03%)	3,599 (7.04%)
North West	189 (2.37%)	93 (2.81%)	208 (2.09%)	542 (2.24%)	216 (3.78%)	1,248 (2.44%)
Elixhauser comorbidity index^1^						
Mean ± SD	1.10 ± 1.76	0.93 ± 1.58	0.88 ± 1.56	0.76 ± 1.48	1.32 ± 1.90	0.91 ± 1.61
Median (IQR)	0.0 (0.0-2.0)	0.0 (0.0-1.0)	0.0 (0.0-1.0)	0.0 (0.0-1.0)	0.0 (0.0-2.0)	0.0 (0.0-1.0)
Elixhauser comorbidity index^1^ (categorized)						
0	4,616 (58.00%)	2,031 (61.38%)	6,376 (63.97%)	16,596 (68.55%)	3,015 (52.83%)	32,634 (63.80%)
1-2	2,005 (25.19%)	808 (24.42%)	2,280 (22.88%)	4,861 (20.08%)	1,452 (25.44%)	11,406 (22.30%)
3+	1,338 (16.81%)	470 (14.20%)	1,311 (13.15%)	2,753 (11.37%)	1,240 (21.73%)	7,112 (13.90%)
Chronic disease						
Asthma	1,731 (21.75%)	615 (18.59%)	1,758 (17.64%)	3,409 (14.08%)	1,026 (17.98%)	8,539 (16.69%)
Chronic obstructive pulmonary disease	4,507 (56.63%)	1,813 (54.79%)	5,124 (51.41%)	10,334 (42.68%)	3,081 (53.99%)	24,859 (48.60%)
Hypertension	5,298 (66.57%)	2,115 (63.92%)	6,124 (61.44%)	14,411 (59.52%)	3,855 (67.55%)	31,803 (62.17%)
Congestive heart failure	1,108 (13.92%)	405 (12.24%)	1,239 (12.43%)	2,586 (10.68%)	1,173 (20.55%)	6,511 (12.73%)

SD, Standard deviation; IQR, Interquartile range; RIO, Rurality Index for Ontario.

1. Comorbidity is based on hospital visits in a 5-year lookback from NSCLC diagnosis. Total score excludes diagnostic codes for lymphoma, metastatic cancer and solid tumours without metastasis.

**Table 2:** Comparison of demographic and general health characteristics according to stage information for non-small cell lung cancer (NSCLC) patients in 2007-2016^1^.

Patient Characteristics	Adjusted Full Model			
	Unstaged vs. Stage I-IV		Unstaged vs. Stage IV	
	RR (95% CI)	p-value	RR (95% CI)	p-value
Year of diagnosis, per 1-year increase	0.91 (0.90-0.92)	<0.001	0.92 (0.91-0.92)	<0.001
Age (categorized)				
60-69 vs. 20-59	1.05 (0.96-1.16)	0.293	1.09 (0.99-1.20)	0.066
70-79 vs. 20-59	1.51 (1.38-1.66)	<0.001	1.57 (1.43-1.71)	<0.001
80+ vs. 20-59	2.87 (2.62-3.15)	<0.001	2.61 (2.39-2.85)	<0.001
Sex				
Male vs. Female	0.95 (0.91-1.00)	0.054	0.93 (0.89-0.98)	0.003
Neighbourhood income quintile				
2 vs. 1 (Lowest)	0.94 (0.87-1.00)	0.064	0.93 (0.87-0.99)	0.026
3 vs. 1 (Lowest)	0.94 (0.87-1.01)	0.083	0.93 (0.87-0.99)	0.035
4 vs. 1 (Lowest)	0.91 (0.84-0.98)	0.012	0.89 (0.83-0.96)	0.002
5 (Highest) vs. 1 (Lowest)	0.89 (0.83-0.97)	0.005	0.90 (0.83-0.97)	0.004
Urban/rural residence				
Urban (RIO<10) vs. Rural (40≤RIO)	0.58 (0.54-0.61)	<0.001	0.58 (0.54-0.61)	<0.001
Sub-urban (10≤RIO<40) vs. Rural (40≤RIO)	0.71 (0.66-0.77)	<0.001	0.73 (0.68-0.78)	<0.001
Elixhauser comorbidity index^2^ (categorized)				
1-2 vs. 0	1.19 (1.12-1.27)	<0.001	1.23 (1.16-1.31)	<0.001
3+ vs. 0	1.49 (1.38-1.60)	<0.001	1.50 (1.40-1.60)	<0.001
Chronic disease				
Asthma, yes vs. no	1.01 (0.95-1.08)	0.765	1.09 (1.03-1.16)	0.004
Chronic obstructive pulmonary disease, yes vs. no	1.02 (0.97-1.07)	0.511	1.13 (1.08-1.19)	<0.001
Hypertension, yes vs. no	0.88 (0.83-0.93)	<0.001	0.92 (0.87-0.97)	0.003
Congestive heart failure, yes vs. no	1.19 (1.11-1.27)	<0.001	1.16 (1.09-1.23)	<0.001

RR, Relative risk; CI, Confidence interval; RIO, Rurality Index for Ontario.

1. Patients with missing responses from specified variables of interest were excluded.

2. Comorbidity is based on hospital visits in a 5-year lookback from NSCLC diagnosis. Total score excludes diagnostic codes for lymphoma, metastatic cancer and solid tumours without metastasis.

**Figure 1:**
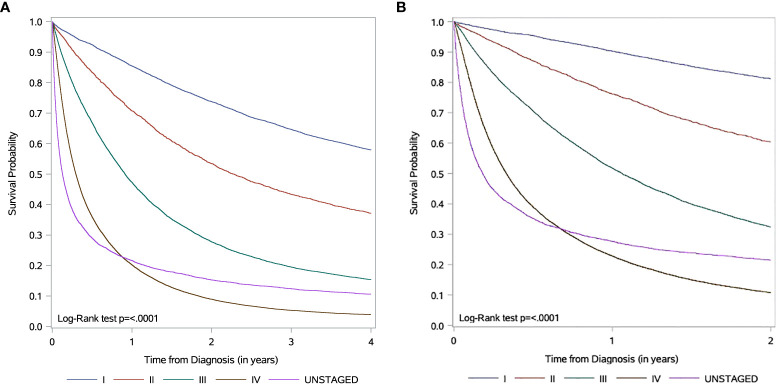
Kaplan-Meier survival curves according to stage information for non-small cell lung cancer (NSCLC) patients in 2007-2016 **(A)** Overall survival. Data is censored 4 years from diagnosis. **(B)** Cancer-specific survival. Data is censored 2 years from diagnosis.

Survival curves are shown in **Figures 1A, B**. For stage III and IV patients, the one-year overall survival (OS) are 47.3% and 20.2% (**Figure 1A**), while the one-year cancer-specific survival (CSS) are 51.8% and 22.8%, respectively (**Figure 1B**). Noticeable in the Kaplan Meier curves is the different shape of the curve for unstaged patients, with a steeper initial drop than stage IV patients, but with a similar one-year survival to stage IV patients (one-year OS: 21.6% vs. 20.2%) and a higher survival in the tail of the curve (four-year OS: 10.6% vs. 3.9%).

## Discussion

In this case study of a large population-based cohort of NSCLC patients, stage data is not missing completely at random. Evaluation of stage-specific health care utilization and outcomes for staged patients, particularly those with stage IV disease, at the population level may thus have a bias as a distinct subset of stage IV patients with rapid death are likely among those without a documented stage in administrative data.

Healthcare utilization differences between staged and unstaged groups was not evaluated in our study. However, it is known that costs (and therefore utilization) vary by lung cancer stage in Canada. A recent study found that unstaged patients with lung cancer had higher costs than stage I and II patients with lung cancer, likely due to the high costs of end-of-life care (6). Treatment receipt for both staged and unstaged groups is delivered based on accepted provincial, national, and international guidelines. These guidelines are based on important prognostic factors not fully available in our cohort, but both groups (staged and unstaged) would have access to fully reimbursed standard of care treatment options. The unstaged group accounted for approximately 11.2% of cases and approximately 12.1% of deaths. These patients have higher comorbidity, rurality, and age than staged patients. It is well known that variation in care and service delivery exists in a single-payer public universal health care system and are associated with patient-level characteristics (7, 8).

Missing stage was also more likely to occur earlier in the study period. Based on the shape of survival curves, we hypothesize that the group without stage data likely represents at least two populations; a rapidly dying advanced cancer cohort dying too quickly to be formally staged or treated in a cancer centre, as well as an earlier stage cohort with better survival with omitted staging due to technical, rather than clinical, reasons. This potential mixture of early and advanced cases argues against simply combining unstaged patients with stage IV patients in studies of stage IV management and outcome.

In population-based studies on palliative systemic therapy utilization in Ontario and possibly other jurisdictions, using a metric of the number of patients who received such therapy divided by all stage IV patients can overestimate utilization (9, 10). This is because the ‘denominator’ of database-recorded stage IV lung cancer may be lower than the ‘true’ number of stage IV patients in the population as a component of patients with true stage IV disease may be missing stage information. In certain populations, like the aged (80+), the bias may be significantly higher, as 40.2% of the unstaged patients were 80+, representing 20.1% of the lung cancers diagnosed in that group.

Cancer stage determination in Ontario is captured by the OCR who receive pathological and clinical (stage assigned by the managing physician) reporting from regional cancer centers across Ontario (11). This process often relies on OCR registrar staff to incorporate and assess clinical, pathological and post-therapy stage information. Other Canadian provincial cancer registries as well as large American cancer registries (National Cancer Database (NCDB) and Surveillance, Epidemiology, and End Results Program (SEER)) have collected stage information following similar processes to Ontario, using trained tumor registrars to abstract specified data elements from patient records in accordance with registry data standards (12).

Our study supports previous findings from other high-income countries of improved (decreasing) rates of missing stage data over time, likely due to improvements in coding standards and cancer registry quality (12, 13). However, as much as stage data capture is improving, it will never be entirely complete due to clinical (e.g., physician failing to assign a category) and data-registry (e.g., miscoded fields) level factors. In the NCDB, high levels of missing data were found for NSCLC and other major cancer sites that also appear not to be missing completely at random. (12). The SEER is also faced with similar challenges with regards to missing data (14). It is highly likely that the COVID-19 pandemic has compromised and continues to affect cancer stage recording and capture, as it has already impacted recent studies (15). Therefore, we expect the trend of decreasing missing cancer data to reverse, and further emphasize the importance of understanding the implications and nature of missing data.

While large databases and staging are helpful in determining real world utilization of palliative systemic therapy and real-world outcomes, there are factors that may bias data collection and interpretation and may lead to over- or under-estimation of treatment utilization. Using only staged patients with stage IV disease to determine palliative systemic treatment utilization in NSCLC may lead to different estimates of utilization in comparison to other methods, such as the ‘lookback’ method from death – which will miss those who have not died, but includes those who receive palliative therapy for unresectable or recurrent disease. Another approach is to look forward from the time of first palliative therapy, which will miss those who receive no palliative therapy, but may include those who had earlier stage disease and subsequently recurred, and those with incurable locally advanced disease (e.g., some stage IIIB). Each of these methods of estimating palliative systemic therapy utilization may lead to different estimates and should be seen as complimentary in determining the real ‘real world’ utilization.

## Conclusion

In this case study, there was evidence that stage data was not missing completely at random. Evaluation of stage-specific health care utilization and outcomes for staged patients with stage IV disease at the population level may have a bias as a distinct subset of stage IV patients with rapid death are likely among those without a documented stage in administrative data.

## Data availability statement

The dataset from this study is held securely in coded form at ICES. While legal data sharing agreements between ICES and data providers (e.g., healthcare organizations and government) prohibit ICES from making the dataset publicly available, access may be granted to those who meet pre-specified criteria for confidential access, available at www.ices.on.ca/DAS (email: das@ices.on.ca). The full dataset creation plan and underlying analytic code are available from the authors upon request, understanding that the computer programs may rely upon coding templates or macros that are unique to ICES and are therefore either inaccessible or may require modification.

## Author contributions

AR: conceptualization, formal analysis, methodology, writing original draft, writing, review and editing. TH: funding acquisition, investigation, writing review and editing. PN: writing, review and editing, data curation, formal analysis, methodology. CG: funding acquisition, writing review and editing. MJ: writing, review and editing, interpretation. All authors contributed to the article and approved the submitted version.
